# Seasonal concentration distribution of PM1.0 and PM2.5 and a risk assessment of bound trace metals in Harbin, China: Effect of the species distribution of heavy metals and heat supply

**DOI:** 10.1038/s41598-020-65187-7

**Published:** 2020-05-18

**Authors:** Kun Wang, Weiye Wang, Lili Li, Jianju Li, Liangliang Wei, Wanqiu Chi, Lijing Hong, Qingliang Zhao, Junqiu Jiang

**Affiliations:** 1 State Key Laboratory of Urban Water Resources and Environment; School of Environment, Harbin Institute of Technology, Harbin, 150090 China; 2 Suzhou Industrial Park Design & Research Co., Ltd, Suzhou, 215000 China

**Keywords:** Environmental sciences, Risk factors

## Abstract

To clarify the potential carcinogenic/noncarcinogenic risk posed by particulate matter (PM) in Harbin, a city in China with the typical heat supply, the concentrations of PM_1.0_ and PM_2.5_ were analyzed from Nov. 2014 to Nov. 2015, and the compositions of heavy metals and water-soluble ions (WSIs) were determined. The continuous heat supply from October to April led to serious air pollution in Harbin, thus leading to a significant increase in particle numbers (especially for PM_1.0_). Specifically, coal combustion under heat supply conditions led to significant emissions of PM_1.0_ and PM_2.5_, especially heavy metals and secondary atmospheric pollutants, including SO_4_^2−^, NO_3_^−^, and NH_4_^+^. Natural occurrences such as dust storms in April and May, as well as straw combustion in October, also contributed to the increase in WSIs and heavy metals. The exposure risk assessment results demonstrated that Zn was the main contributor to the average daily dose through ingestion and inhalation, ADD_Ing_ and ADD_inh_, respectively, among the 8 heavy metals, accounting for 51.7–52.5% of the ADD_Ing_ values and 52.5% of the ADD_inh_ values. The contribution of Zn was followed by those of Pb, Cr, Cu and Mn, while those of Ni, Cd, and Co were quite low (<2.2%).

## Introduction

Outdoor air pollution caused by unreasonable energy consumption, rapid industrialization and urbanization ranks among the most critical environmental issues in China^1,2^. Specifically, atmospheric particulate matter (PM), known as PM_1.0_, PM_2.5_ and PM_10_, has drawn much attention because of its acute and chronic effects on human health (such as cardiovascular and lung cancer mortality)^3–5^. Traditionally, a decrease in the aerodynamic diameter has always led to a significant increase in the chemical reactivity of PM for virus and trace heavy metal adsorption, undoubtedly enhancing the toxicity/health risk of those atmospheric particulates^6,7^.

PM consists of a mixture of chemical components, which may be directly emitted from primary sources or formed through complex atmospheric processes^8^. Generally, primary PM is always released into the atmosphere via wind, combustion processes and anthropogenic activities (including fossil/agricultural energy combustion, industrial processes, construction, etc.), whereas photochemical reactions (O_3_ oxidation, solar irradiation) and other chemical processes lead to the significant formation of secondary PM, such as sulfate, nitrate, ammonium, and secondary organic aerosols (SOAs)^9^. Due to the considerable dependence on fossil fuel, air pollution in northern China is more severe than that in the southern regions, especially in the heating season^10,11^. A report by the Ministry of Environmental Protection of China revealed that 33 cities were seriously polluted in January 2013, and the most polluted regions were mainly located in the northern region of China, including the Beijing-Tianjin-Hebei region, central and southern Hebei, southwestern Heilongjiang, western Shangdong, and central Liaoning and Henan provinces^12,13^. Specifically, primary emissions originating from fossil/agricultural waste combustion, power generation, industrial processes, and road traffic were the main reasons for atmospheric pollution^1,14^.

Inorganic water-soluble ions (WSIs) are among the predominant chemical constituents of the atmospheric pollutants of PM, which play a substantial role in the Earth’s radiation balance and are related to atmospheric particle formation, growth and transformation^15^. Recent publications by Sun *et al*. and Cheng *et al*. demonstrated that as much as 45.0%–55.5% of atmospheric PM was attributed to WSIs^16,17^, in which the inorganic WSIs SO_4_^2−^, NO_3_^−^, and NH_4_^+^ have a substantial effect on the atmospheric extinction coefficient and subsequently decrease urban visibility^18^. In addition, heavy metals are the essential toxic components of PM, and these nonbiodegradable elements can severely affect human health via three routes of exposure: food intake, skin contact and inhalation^19,20^. Among these hazardous heavy metals, Cu, Cr, Fe, Ni, Pb, Zn, V, Cd, and Mn, are the metals of the most immediate concern because of their detection frequency. Exposure to these metals has been associated with cardiovascular diseases, cancer and many other adverse health effects^21^, and their specific effects within different age groups varied widely^22^. Since the mobility, stability and solubility of heavy metals depend on their speciation characteristics^23^, sequential fractionation technology has been used for toxicity analysis^24^. However, few studies have addressed the association between the concentrations of aerosolized metallic elements in PM_1.0_ and PM_2.5_ and the adverse health impacts on local residents, especially the effect of heavy metal species from the long-term heat supply.

Here, we examined the total mass and species distribution of typical heavy metals and WSIs in PM_2.5_ and PM_1.0_ in Harbin and analyzed the variation in these species throughout the year. The concentrations in the periods with and without heat supply were compared. Moreover, the possible health risks posed by heavy metals, especially different metal species, when children and adults were exposed to them were assessed.

## Results and discussion

### Mass concentration variations in the PM_2.5_ and PM_1.0_ of Harbin

**Figure 1:**
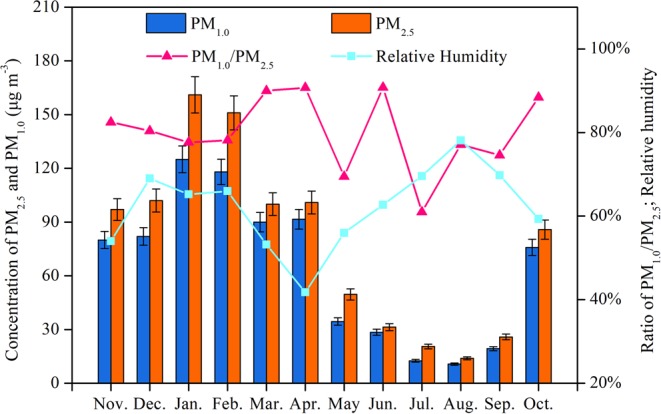
Variation in the relative humidity and mass concentrations of PM_1.0_ and PM_2.5_ collected in different months from Nov. 2014 to Oct. 2015 in Harbin.

The mass concentrations of PM_2.5_ and PM_1.0_, shown in Fig. 1, of the samples in the port of Harbin were analyzed. Overall, the concentration distribution trend of PM_1.0_ was similar to that of PM_2.5._ Both exhibited a plateau during the periods of Oct. to Apr. The average mass concentrations of PM_2.5_ and PM_1.0_ ranged from 14.1 to 161.4 μg·m^−3^ and 11.2 to 125.3 μg·m^−3^, respectively, with the highest value found in January and the lowest in August. Generally, the average PM_2.5_ value in Oct., Nov., Dec., Jan., Feb., Mar. and Apr. was 1.14–2.15 times greater than the threshold limit values (75 μg/m^3^ for PM_2.5_) in the Air Quality Standard of China.

Because PM_1.0_ has a larger specific surface area than PM_2.5_, toxic pollutants can be more easily adsorbed onto PM_1.0_ and pose a greater threat to human health^20,25^. Monthly distributions of PM_1.0_/PM_2.5_ (Fig. 1) were calculated and varied widely during the continuous 12 months of detection. A higher PM_1.0_/PM_2.5_ ratio (0.61–0.91) implied that fine particle PM_1.0_ was the predominant component of the PM. In addition, a higher PM_1.0_/PM_2.5_ ratio for the heat supply periods (average ratio of 0.832) than the PM_1.0_/PM_2.5_ ratio for the months with no heat supply (average ratio of 0.769) was related to the coal combustion and pollutants produced from the physical/chemical reactions of atmospheric ions.

### Monthly variation in particle number concentrations

The variation in the particle numbers of PM_1.0_ was similar to that of PM_2.5_; thus, the particle distribution of PM_1.0_ was selected as an example and is discussed here. Overall, the particle numbers of PM_1.0_ were much higher in the heat supply period than in the period with no heat supply (see Supplementary Fig. S1), with a maximum value (in particle number/cm^3^) of 50714 in December and a minimum in August (9312). Moreover, the difference between the maximum value and minimum value of the particle number, observed in the heat supply periods, is quite significant, whereas that in the period with no heat supply is not significant (42367 *vs* 6188). The main reason for this finding might be that (1) the higher humidity observed in the period with no heat supply than in the period with heat supply enhanced the precipitation and settlement of PM and (2) the continuous heat supply in winter led to a more significant particulate emission. Similar observations were reported by Jones and Harrison^26^. The ratio of the mass concentration/particle number of PM_1.0_ for the samples observed from the heat supply period is lower than that from the months with no heat supply (Supplementary Table S1), implying that the predominant particles in the collected PM_1.0_ in the heat supply period existed as fine particles (smaller average diameter), ascribed to the effective removal of the larger particles by dust removal equipment.

### Concentration distribution of inorganic/metal elements and WSIs in PM_1.0_ and PM_2.5_ fine particles

To detect the elements trapped on the fine particles, the concentrations of inorganic elements, as well as WSIs within PM_2.5_ and PM_1.0_ obtained from different months, were analyzed, and the distribution of the typical trace elements of PM_1.0_ was selected as an example and discussed (PM_1.0_ in Table 1 and PM_2.5_ in Table S2). Generally, the monthly concentration variation in different trace elements could reveal the origin of those pollutants and the possible sources of those elements^27,28^.

**Table 1:** Concentrations of inorganic/metal elements and WSIs in PM_1.0_ in different months from Nov. 2014 to Oct. 2015 (unit: μg/m^3^).

		Dec	Jan	Feb	Mar	Apr	May	Jun	Jul	Aug	Sep	Oct	Nov
Al	inorganic elements	0.63	0.48	1.80	0.70	1.28	0.69	0.29	0.16	0.13	0.24	0.29	0.29
Fe		1.42	1.37	2.82	1.28	2.10	4.00	3.26	3.14	0.76	1.85	1.40	0.76
S		5.25	12.34	8.15	4.43	2.74	3.91	2.43	1.95	1.25	2.79	3.80	4.39
Si		0.58	5.17	3.15	0.91	0.86	0.66	0.48	0.84	0.68	0.74	0.71	0.71
Zn		0.53	0.45	0.43	0.73	0.58	0.28	0.28	0.38	0.22	0.34	0.55	0.74
Ti		0.30	0.39	0.40	0.39	0.41	0.51	0.32	0.46	0.32	0.44	0.46	0.40
Pb		0.18	0.14	0.20	0.24	0.11	0.06	0.12	0.08	0.07	0.09	0.13	0.09
As		0.07	0.03	0.02	0.02	0.04	0.04	0.00	0.00	0.00	0.01	0.01	0.02
Ba		0.06	0.05	0.22	0.14	0.10	0.09	0.02	0.03	0.02	0.03	0.04	0.02
Mn		0.05	0.04	0.06	0.04	0.12	0.06	0.09	0.05	0.04	0.04	0.04	0.04
Cu		0.05	0.05	0.07	0.06	0.12	0.04	0.02	0.02	0.03	0.02	0.14	0.04
Cr		0.07	0.10	0.18	0.11	0.05	0.30	0.32	0.42	0.12	0.21	0.15	0.11
Sr		0.02	0.02	0.06	0.04	0.03	0.03	0.01	0.01	0.01	0.01	0.01	0.01
Ni		0.01	0.04	0.02	0.02	0.04	0.02	0.02	0.05	0.02	0.02	0.03	0.03
Ca		8.63	6.28	8.16	9.09	15.28	9.12	5.20	5.36	2.98	4.21	5.77	5.97
K		2.06	1.22	3.32	3.03	1.59	1.84	1.11	1.15	0.75	1.88	4.36	4.95
Mg		0.76	0.54	1.20	0.89	1.54	1.17	0.45	0.85	0.32	0.55	0.76	0.72
Na		12.13	9.87	8.18	7.26	11.54	13.26	5.95	7.29	4.66	10.19	10.53	7.17
Ca^2+^	WSIs	2.61	6.26	2.94	2.72	2.85	1.45	1.07	1.01	0.64	0.96	1.78	1.69
K^+^		1.14	0.68	1.20	1.42	1.05	0.77	0.60	0.45	0.37	0.63	1.71	1.84
Mg^2+^		0.11	0.33	0.66	0.37	0.27	0.17	0.13	0.18	0.06	0.15	0.29	0.15
Na^+^		1.35	0.29	1.61	0.56	4.24	2.30	1.33	2.01	0.69	2.31	3.19	1.16
F^−^		0.19	0.24	0.49	0.35	0.30	0.06	0.03	0.14	0.04	0.06	0.22	0.25
NH_4_^+^		6.6125	7.024	14.25	6.2425	4.1322	3.8895	2.2441	3.7991	2.7721	3.5617	4.0365	3.95
Cl^−^		2.7185	3.275	8.1525	6.6005	3.29	1.1883	0.778	0.3789	0.2883	0.132	2.5133	3.8765
SO_4_^2−^		9.3525	28.206	13.72	22.337	9.007	5.146	4.8	1.6506	1.5023	3.104	4.1973	4.4915
NO_3_^−^		6.4345	14.536	15.863	6.1905	4.9025	2.94	3.9575	1.5781	1.2122	0.475	6.4512	8.504
Σ		63.32	99.42	97.33	76.18	68.57	54.01	35.32	33.44	19.95	35.04	53.57	52.38

As shown in Table 1, the maximum concentration of the majority of inorganic/metal elements in Harbin was found in the heat supply period, except for Fe, Ti, Mn and Cr. In brief, the concentrations of Cu, K, and Zn in the fine particles were highest in Oct. and Nov., and As, S, Al, Ba, Sr and Si occurred in Dec., Jan. and Feb., while Ca, Mg, Pb and Mn were found in Mar. and Apr. Ca exhibited the highest concentration among all 18 non-WSIs, which is related to its wide application in building materials. Similarly, elemental Na ranked as the second-highest emission in particulates during the whole year, mainly originating from natural emissions and human activities. By contrast, the existence of Pb and Zn, specifically in summer, was partially ascribed to automobile exhaust (Pb in the heat supply period was also related to coal combustion). In addition, the continuous heat supply in Dec., Jan. and Feb. contributed to the higher emissions of S, As, Si to the atmosphere, which was mainly ascribed to coal combustion.

**Figure 2:**
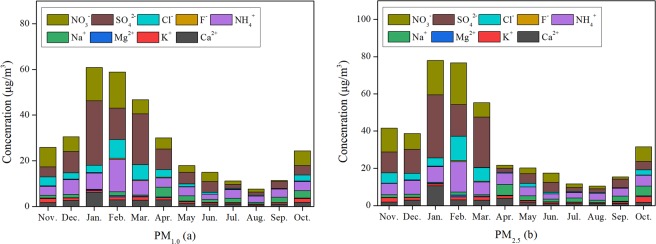
Concentrations of WSIs in PM_1.0_ (**a**) and PM_2.5_ (**b**) in different months from Nov. 2014 to Oct. 2015.

Among all 9 selected WSIs, the secondary atmospheric ions of SO_4_^2−^, NO_3_^−^, NH_4_^+^ were predominant in the fine particles, which originated mainly from physical/chemical reactions^29^. Generally, WSIs were higher in the heat supply period than in the seasons with no heat supply, both for PM_1.0_ and PM_2.5_ (Fig. 2). For example, the mass concentration of SO_4_^2−^ reached its maximum value in Jan., with an average value of 28.21 μg/m^3^ for PM_1.0_ and 33.88 μg/m^3^ for PM_2.5_. Similarly, the highest values of NO_3_^−^ and NH_4_^+^ were observed in Feb., exhibiting maximum concentrations (in μg/m^3^) of 15.86 and 14.25 for PM_1.0_, respectively. Since the atmospheric condition of the inland city Harbin was insignificantly affected by sea salt, the WSIs of Na^+^, Mg^2+^, and Cl^−^ in the collected particles originated from industrial emissions and natural sources^30,31^. Na^+^ ions, mainly originating from crustal sources in Harbin, exhibited the highest value of 4.24 μg/m^3^ in Apr., ascribed to the resuspended road dust, soil dust, and construction dust due to the strong wind in spring (average speed of 3.7 m/s)^17^. Correspondingly, the highest concentration of K^+^ in Nov. for PM_1.0_ (1.84 μg/m^3^) is mainly ascribed to straw combustion in Heilongjiang Province in autumn^32^, whereas Feb. had the highest levels of Cl^−^ (8.15 μg/m^3^ for PM_1.0_). The average Cl^−^ in PM_1.0_ during the periods with and without heat supply was 4.65 μg/m^3^ and 0.88 μg/m^3^, respectively, which is usually considered to be from coal combustion^33^.

The seasonal percentage distribution of 18 elements for particles PM_1.0_ and PM_2.5_ varied widely (in Fig. S2). Since the concentrations of Zn, Ti, Pb, As, Cu, Cr and Ni in the chemical composition of PM_1.0_/PM_2.5_ are quite low, the sum of those proportions are defined as other concentrations. K mainly originated from the burning of biomass; thus, K was higher (>14%) in autumn for both PM_1.0_ and PM_2.5_ than in the other three seasons. S mainly originated from coal combustion and exhibited the highest value in winter. For comparison, Ca was highest in spring, which is related to the windy weather and dust emissions from building materials. Although Si basically originates from natural sources, coal combustion in winter contributed a significant amount of emissions into the atmosphere; thus, a higher concentration in winter than in the other seasons was finally observed. As shown in Table S3, the majority of the elements in PM_1.0_ and PM_2.5_ existed as inorganic elements regardless of the seasonal variation (≥58.0%), except for S and Si in spring. It should be noted that most of the inorganic elements in PM_1.0_ are highly enriched, demonstrating that the majority of those inorganic particles were attached to PM_1.0_, and the seasonal variation insignificantly affected their distribution.

### Source apportionment analysis of PM_2.5_ and PM_1.0_ in different seasons

Potential sources of PM_2.5_ and PM_1.0_ in Harbin were evaluated based on the chemical constituent analysis of 150 PM_2.5_ and PM_1.0_ samples via principal component analysis-multiple linear regression (PCA-MLR) model utilization. In total, 6 components were resolved from the PCA according to Bhuyan *et al*.^34^ and Cusack *et al*.^35^, in which principal factors with eigenvalues >0.8 were chosen by extracting the eigenvalues and eigenvectors. Factor 1, with higher loadings of Cu, Fe, Mn, Ni and Pb, was typically applied to explain the characteristics of road dust. Factor 2 explained 17.1% of the variance with high loadings of NO_3_^−^, SO_4_^2−^, WSOC and NH_4_^+^, indicating the influence of secondary aerosol sources over the site. Factor 3 represented the construction materials, with high loadings of Ca, Mg, Na ^+^ and Cl^−^, closely related to the cement manufacturing processes and to the end usage at construction sites. Factor 4 showed high loadings of SO_4_^2−^, NO_3_^−^, Na^+^, NH_4_^+^, K^+^ and organic carbon (OC), indicating combustion sources, biomass burning (SO_4_^2−^, NO_3_^−^ and K^+^) and vehicular emissions (OC, NO_3_^−^). Factor 5 was characterized by high loadings of Al, Na, K and Si, which points to crust/soil emissions. Factor 6, characterized by a high F^−^ loading, is related to fossil fuel (especially coal) combustion emissions.

**Figure 3:**
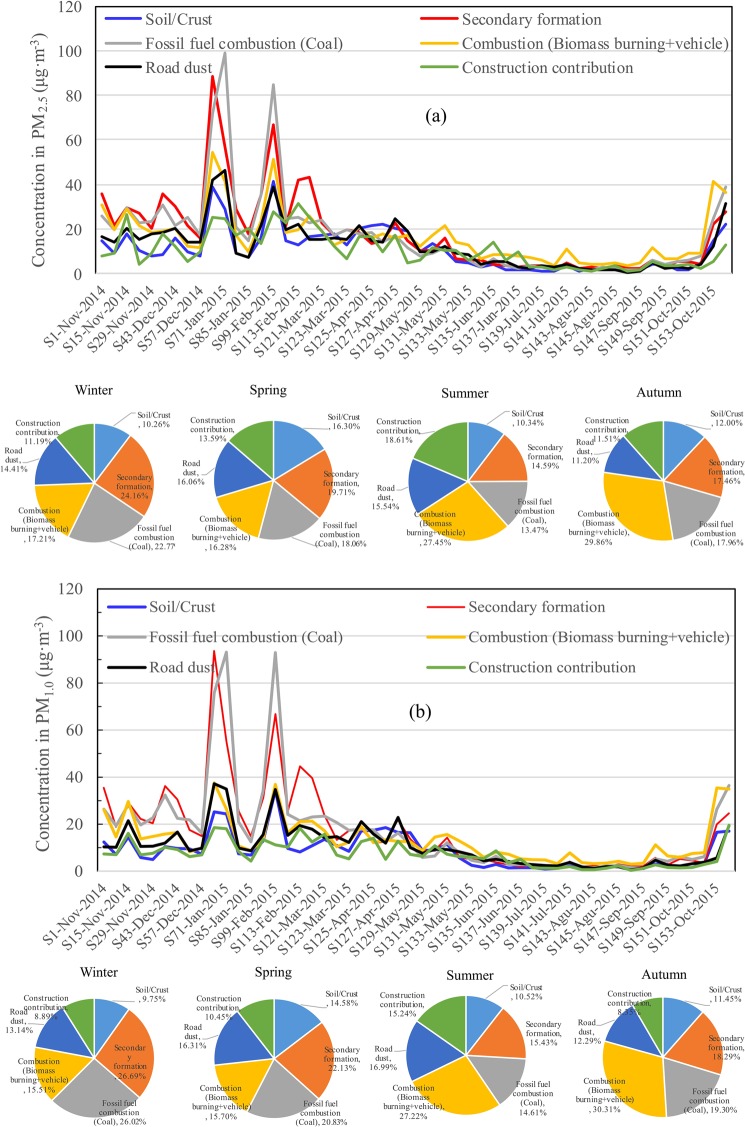
Time-evolved source contributions to different PM_2.5_ (**a**) and PM_1.0_ (**b**) samples obtained from the PCA-MLR results (μg·m^−3^).

The contributions of each source/group of sources were predicted according to Bhuyan *et al*.^34^ and are listed in Fig. 3. Overall, the percentage contribution of Factor 1, road dust, changed insignificantly with seasonal variations and was the highest in spring and lowest in autumn. Similarly, Factor 5 of crust/soil emissions accounted for as much as 16.1% of the PM_2.5_ pollution in spring and was the lowest in winter (11.2%). For comparison, both the secondary formation (Factor 2) and fossil fuel combustion (Factor 6) exhibited the highest percentage contribution in winter (24.2% and 22.8%, respectively), followed by spring, whereas it was lowest in summer, ascribed to the heat supply from Oct. to Apr. for Harbin. In addition, the highest percentage contribution of biomass burning and vehicle emissions in autumn (29.9%) was related to the burning of straw, as well as the presence of 1.8 million automotive vehicles in Harbin City. The above results were similar to the previous observations of Shi *et al*.^36^, who found that the top five significant contributors to PM_10_ in Chengdu were vehicle exhaust (28.71%), coal combustion (24.45%), resuspended dust (19.24%), secondary sulfate+nitrate (18.20%), and soil dust (16.53%). Similarly, the observations of Bhuyan *et al*.^34^ revealed that the most dominant sources of the mid-Brahmaputra Valley of India were combustion sources, followed by road dust, construction dust and soil/crust input. The seasonal percentage contributions of the six sources of PM_1.0_ were similar to those of PM_2.5_, in which the contributions of secondary formation and fossil fuel combustion were higher for PM_2.5_ than for PM_1.0_, whereas soil/crust, combustion (biomass burning + vehicles), road dust and construction contributions were lower.

Overall, the percentage contributions of the six main PM_2.5_ sources of Harbin in the heat supply period were distributed as secondary formation (22.0%) > fossil fuel combustion (21.2%) > combustion (biomass burning + vehicles) (17.6%) > road dust (14.6%) > soil/crust (12.5%) > construction contribution (12.0%). By contrast, the contributions of secondary formation and fossil fuel combustion (coal) decreased to 16.1% and 15.1%, respectively, in the periods with no heat supply. Based on the above analysis, we conclude that coal combustion and secondary aerosols were the main sources of PM pollution in Harbin, especially in seasons with heat supply. Moreover, PM_1.0_ is the most important fine PM emitted for most pollution sources. Thus, prevention and control measures should be urgently developed.

**Figure 4:**
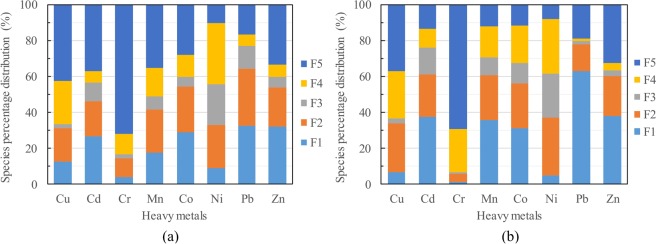
Species distribution of eight heavy metals in PM_2.5_ in heat supply periods (**a**) and in periods with no heat supply (**b**).

Speciation characteristics of heavy metals in PM_2.5_. Excess heavy metals in PM_2.5_, especially the weakly bonded exchangeable fractions, endanger natural systems and human health^37^. Speciation distributions of heavy metals in PM_2.5_ were characterized, and the corresponding speciation information is given in Fig. 4. Overall, the majority of the eight selected metals preferentially existed as a residual fraction (mineral crystal lattice), followed by water- and acid-exchangeable phases, while the reducible, oxidizable fractions had substantially lower contents. Briefly, the residual fraction of Cr, accounting for as much as 71.5% of the bulk Cr, was the highest among all eight metals, followed by Cu (41.1%), Zn (33.1%), Cd (31.1%), Mn (29.5%), Co (24.0%), Pb (17.0%), and elemental Ni (9.6%). For comparison, the water-exchangeable fraction of eight elements decreased in the order of Pb(40.3%) > Zn(33.6%) > Cd(29.7%) > Co(29.4%) > Mn(22.3%) > Cu (11.3%) > Ni(8.0%) > Cr(3.2%). Overall, Cd, Co, Pb, Mn and Zn exhibited a higher percentage distribution in the water/acid-exchangeable fractions (>46.4%) than in the other fractions.

According to Sabiene *et al*.^38^, residual, oxidizable and reducible heavy metals are quite refractory and are generally recognized as a stable fraction due to their weak bioavailability and low solubility, while water/acid-exchangeable heavy metals are defined as a labile fraction^39^. As shown in Fig. 4, Pb was the most labile element among all eight heavy metals in PM_2.5_, accounting for 40.3% as water-exchangeable forms and 27.7% as acid-exchangeable forms. Zn was the second-most labile heavy metal in PM, exhibiting a relatively high percentage distribution of water- and acid-exchangeable fractions (33.6% and 22.1%, respectively). By contrast, Ni, Cu and Cr showed a lower percentage of labile fractions (≤34.1%). Overall, Cr was the most refractory metal in PM_2.5_ (3.2% for water- and 9.2% for acid-exchangeable Cr). Based on the binding strength and solubility in different geochemical fractions of heavy metals, the potential toxicity of different heavy metal fractions is expected to decrease in the following order: exchangeable (F_1_) > carbonates (F_2_) > reducible (F_3_) > oxidizable (F_4_) > residual metal phase (F_5_)^40^. Thus, we concluded that the elements Ni, Cu and Cr in PM_2.5_ had a low hazard potential. For comparison, elemental Pb exhibited a converse trend, with the highest percentage ratio for F1 and F2 (40.3% and 27.7%, respectively).

Metal fractionation occurring in PM_2.5_ and PM_1.0_ is, in turn, likely to influence metal toxicity^41^. Toxicities of heavy metals in PM can be calculated according to the distribution of the fractional species. The equations for the risk assessment code (RAC) calculation can be summarized as follows:

1 $${\rm{RAC}}=\frac{{F}_{1}+{F}_{2}}{{F}_{1}+{F}_{2}+{F}_{3}+{F}_{4}+{F}_{5}}\times 100 \% $$

2 $${C}_{{\rm{f}}}=({F}_{1}+{F}_{2}+{F}_{3}+{F}_{4})/{F}_{5}$$

where RAC is the risk assessment code (%) of heavy metal pollution. A value of RAC < 1% usually demonstrates no risk; values of 1%≤RAC < 10% and 10%<RAC < 30% refer to low and medium risk, respectively; and an RAC ≥ 30% indicates a high risk, especially when RAC > 50% (very high risk). *F*_1_, *F*_2_, *F*_3_, *F*_4_ and *F*_5_ represent the percentage distributions of the water-, acid-, reducible-, oxidizable- and residual heavy metal fractions in PM_2.5_, respectively_._ C_f_ refers to the pollution coefficient.

**Figure 5:**
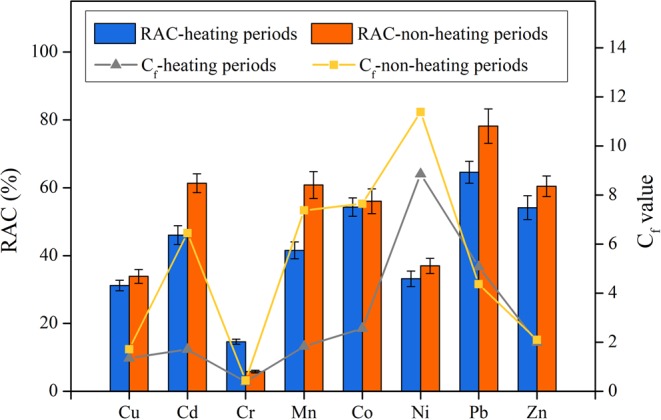
Risk assessment code and pollution coefficient distribution of eight heavy metals in PM_2.5_.

As shown in Fig. 5, the results obtained from the RAC calculation demonstrated that Pb, Zn, Co and Cd in PM_2.5_ were at a very high-risk level, which decreased in the order of Pb (68.0%)>Zn (55.7%)>Co (54.7%)>Cd (50.2%). For comparison, the elements Mn (46.4%), Ni (34.1%) and Cu (31.9%) were at a high level, whereas the RAC of elemental Cr was at a medium risk level (RAC = 12.4%). The distribution trend of *C*_f_ was quite different from that of RAC and decreased in the order of Ni(9.39)>Pb(4.88)>Co(3.18)>Mn(2.39)>Cd(2.23)>Zn(2.02)>Cu (1.43)>Cr(0.40). Although Ni was at a low concentration level in PM_2.5_, its high RAC pollution potential implied that we should be highly concerned. In addition, the higher toxicities of Cd, Co, Pb, and Zn demonstrated that the emissions from coal combustion, the electroplating industry, metallurgy, the chemical industry and private cars in Harbin City should definitely be controlled, especially in the winter season.

The distribution of the fractional species of heavy metals obtained from the periods with no heat supply was similar to the distribution in the heat supply periods (seen in Fig. 5). Specifically, the RAC of Cu, Co, Ni and Zn exhibited a slight increase during the periods with no heat supply compared to that during the heat supply period (<12%), and that of Cd, Mn and Pb increased significantly (41.5%-64.6%). The higher concentration of Cr observed in the heat supply period (listed in Table 1), as well as the corresponding higher RAC value, implied that the hazardous element Cr within the particles should be given more attention. All the tested heavy metals except Pb exhibited an increased C_f_ value (especially for Cd, Mn and Co) during the period with no heat supply, implying that coal combustion during heat supply periods contributed to the emission/production of the residual fraction of heavy metals.

### Risk assessment

A carcinogenic and noncarcinogenic risk assessment for Cr, Ni, Cd, Co, Pb, Cu, Zn, and Mn in PM_2.5_ samples was performed using the health risk assessment model of the US EPA, and three exposure pathways, namely, ingestion, inhalation, and dermal contact, were separately studied (Table 2). In general, the hazard quotient results demonstrated that the three exposure pathways had the same trends of ingestion> dermal contact> inhalation, implying that ingestion was the most health-threatening exposure route for heavy metals in PM_2.5_ in Harbin. Moreover, children suffered from a higher risk from ingestion and dermal contact from PM_2.5_ than adults. Specifically, the ADD_Ing_ values for children were 7.23 and 8.21 times higher than the values for adult males and females, respectively, indicating that children not only experienced a higher carcinogenic and noncarcinogenic risk but were also more vulnerable to that risk than adults. Similarly, the ADD_Derm_ for children was 5.63 and 5.82 times higher than the ADD_Derm_ for adult males and females, respectively. Conversely, the ADD_Inh_ values obtained for children were 48.4% and 41.9% lower than the values for adult males and females, respectively.

**Table 2:** Ingestion, inhalation, and dermal contact exposure risks of eight heavy metals in PM_1.0_ and PM_2.5_ in Harbin City.

Elements	ADDIng (mg‧kg^−1^·d^−1^)			ADDderm (mg·kg^−1^·d^−1^)			ADDInh (mg·kg^−1^·d^−1^)		
	Children	Females	Males	Children	Females	Males	Children	Females	Males
Cr	1.33 × 10^–2^	1.83 × 10^–3^	1.77 × 10^–3^	7.16 × 10^–5^	4.88 × 10^–5^	5.23 × 10^–5^	9.26 × 10^–8^	1.92 × 10^–7^	2.22 × 10^–7^
Ni	2.62 × 10^–3^	3.61 × 10^–4^	3.50 × 10^–4^	1.41 × 10^–5^	9.65 × 10^–6^	1.03 × 10^–5^	1.83 × 10^–8^	3.79 × 10^–8^	4.38 × 10^–8^
Cd	1.21 × 10^–3^	1.67 × 10^–4^	1.61 × 10^–4^	6.52 × 10^–7^	4.45 × 10^–7^	4.77 × 10^–7^	8.44 × 10^–9^	1.75 × 10^–8^	2.20 × 10^–8^
Co	5.71 × 10^–4^	7.87 × 10^–5^	7.62 × 10^–5^	3.08 × 10^–6^	2.10 × 10^–6^	2.25 × 10^–6^	3.99 × 10^–9^	8.25 × 10^–9^	9.54 × 10^–9^
Pb	2.14 × 10^–2^	2.95 × 10^–3^	2.56 × 10^–3^	4.62 × 10^–3^	7.89 × 10^–4^	7.56 × 10^–4^	1.50 × 10^–7^	3.10 × 10^–7^	3.58 × 10^–7^
Cu	1.13 × 10^–2^	1.56 × 10^–3^	1.35 × 10^–3^	2.44 × 10^–4^	4.17 × 10^–5^	3.99 × 10^–5^	7.90 × 10^–8^	1.64 × 10^–7^	1.89 × 10^–7^
Zn	6.73 × 10^–2^	9.28 × 10^–3^	8.05 × 10^–3^	1.45 × 10^–3^	2.48 × 10^–4^	2.38 × 10^–4^	4.70 × 10^–7^	9.74 × 10^–7^	1.13 × 10^–6^
Mn	1.05 × 10^–2^	1.45 × 10^–3^	1.26 × 10^–3^	2.27 × 10^–4^	3.88 × 10^–5^	3.72 × 10^–5^	7.35 × 10^–8^	1.52 × 10^–7^	1.76 × 10^–7^
Σ	1.28 × 10^–1^	1.77 × 10^–2^	1.56 × 10^–2^	6.64 × 10^–3^	1.18 × 10^–3^	1.14 × 10^–3^	8.96 × 10^–7^	1.85 × 10^–6^	2.14 × 10^–6^

As shown in Table 2, Zn was the main contributor to the ADD_Ing_ and ADD_inh_ among the 8 heavy metals, accounting for 51.7–52.5% of the bulk ADD_Ing_ values for children, males and females; the next most significant contributors were Pb, Cr, Cu and Mn, with values ranging from 16.4–16.7%, 10.3–11.4%, 8.7–8.8%, and 8.1–8.2%, respectively, while the ADD_Ing_ values of Ni, Cd, Co were quite low (<2.2%). Similarly, Zn was also the predominant source of the ADD_Inh_ risk and accounted for 52.5% of the bulk ADD_Inh_ risk. Pb was the second highest risk source, and Cr ranked third, accounting for 16.7% and 10.3%, respectively. Cu and Mn were also important sources of the ADD_Inh_ risk and contributed 8.8% and 8.2% of the bulk risk, respectively. For comparison, Pb played the most important role in ADD_Derm_ risk and accounted for 66.5–69.7% of the bulk ADD_Derm_ risk, followed by Zn (20.9–21.9%), while the ADD_Derm_ risk of Cr, Ni, Cd, Co, Cu and Mn was quite low. In addition, Pb, Cu, Zn and Mn exhibited a higher ADD_Derm_ risk for adults than for children, while the ADD_Derm_ of Cr, Ni, Cd, and Co was significantly lower (especially for Cr and Ni). From the above, we can conclude that Zn and Pb in the PM_2.5_ pollutants of Harbin City should be preferentially considered for their high environmental risk and RAC value; Cr, Cu, and Mn should be controlled first, while the environmental risk of Ni, Cd and Co was quite low.

Because the toxicity of the heavy metals was significantly affected by the fractional species distribution of the heavy metals, the risk assessments were further combined and analyzed with the fractional results. Compared to the periods with no heat supply, the RAC value of Cr, as well as the C_f_ value of Pb, both exhibited a noticeable increase during the heat supply periods; thus, the pollution of Cr and Pb from coal combustion in heat supply periods should be of great concern. In contrast, Zn, Cu, Co and Mn from street dust should also be at a high level of concern in periods with no heat supply for controlling health risks^42^ due to their high C_f_ value and high ecological risk.

## Conclusions

1. The continuous heat supply from Oct. to Apr. led to serious air pollution in Harbin. The tremendous amount of emissions of the fine particle PM_1.0_ during the heat supply period led to a higher PM_1.0_/PM_2.5_ value of 0.832 than that during the months with no heat supply. The PM_1.0_ particle number was highest in Dec. (50714/cm^3^), which was 4.91 times higher than the minimum value found in August.
2. The concentrations of the majority of the 9 selected WSIs in PM_1.0_ and PM_2.5_ were higher in the heat supply period than in the seasons with no heat supply. Specifically, the WSI of SO_4_^2−^ reached the maximum value in Jan.; NO_3_^−^, NH_4_^+^ and Cl^−^ in Feb.; Ca^2+^, K^+^ and F^−^ in Apr.; and Na^+^ in May.
3. The percentage contributions of the six main PM_2.5_ sources in Harbin in the heat supply period were distributed as secondary formation (22.0%)>fossil fuel combustion (21.2%)>combustion (biomass burning+vehicles) (17.6%)>road dust (14.6%)>soil/crust (12.5%)>construction (12.0%). The contributions of secondary formation and coal combustion decreased to 16.1% and 15.1%, respectively, in the periods with no heat supply.
4. The RAC results demonstrated that Pb, Zn, Co and Cd in PM_2.5_ created very high risk levels; Mn, Ni and Cu created a high risk level, while Cr was at a medium risk level (highest residual fraction of 71.5%). The heat supply increased the RAC value of Cr and the C_f_ value of Pb. Ingestion was the most health-threatening exposure route for heavy metals in PM_2.5_ in Harbin. Zn was the main contributor to the ADD_Ing_ and ADD_inh_ among the 8 heavy metals, followed by Pb, Cr, Cu and Mn.

## Materials and methods

### Description of the city of Harbin

Harbin, one of the ten most populated cities in China, is the capital of Heilongjiang Province and is the commercial, industrial, and transportation center of Northeast China. The city is situated on the Songnen Plain, surrounded by mountain chains (the Lesser Khingan Mountains, the Changbai Mountains and the Higher Lesser Khingan Mountains in the north, east and west, respectively, to form a low-lying center), so special geographical and meteorological factors slow the wind speed. The mean annual temperature in Harbin is 3.5 °C; thus, heat supply is necessary during the period of Oct. 20 to Apr. 20^43^. In 2015, the air quality of Harbin had levels of 24.1% excellent, 38.4% good, 19.7%, 5.5%, 8.5% and 3.8% light, moderate, severe and serious pollution, respectively (http://www.hljdep.gov.cn/hjgl/hjjc/hjzljc/2016/01/11891.html).

### PM sample collection and sampling site description

**Figure 6:**
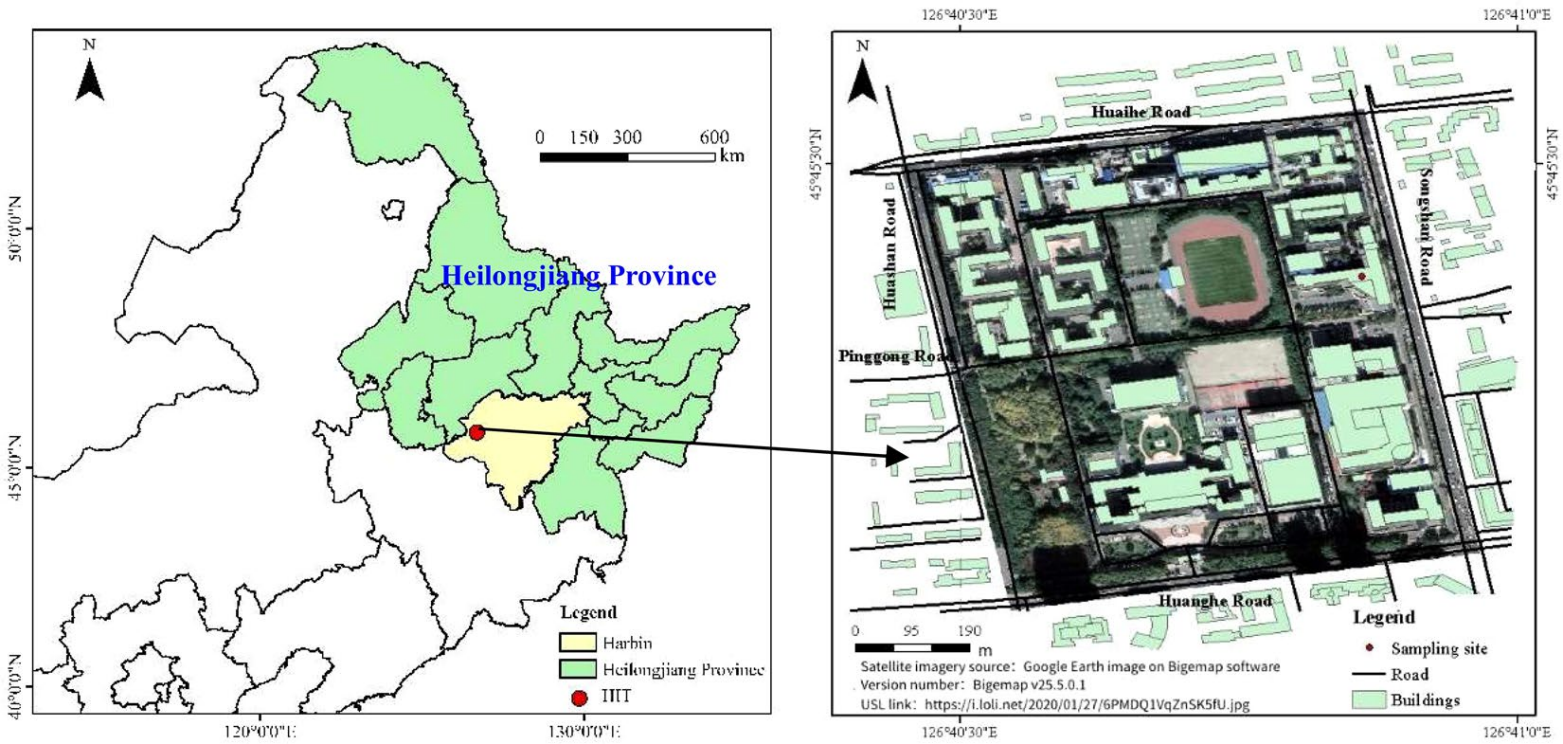
The location of the study area in China and the sampling sites (satellite imagery was obtained from Google Earth 3D image on Bigemap software (version number v25.5.0.1,URL link:https://i.loli.net/2020/01/27/6PMDQ1VqZnSK5fU.jpg)).

As shown in Fig. 6, atmospheric PM was sampled on the roof of the School of Environment (14 m high) of the Harbin Institute of Technology (HIT) (longitude 126.691°E and latitude 45.763°N). The sampling site was close to Songshan Road and Huanghe Road (with dense vehicular traffic) and surrounded by numerous commercial centers, residential quarters, and power plants. Approximately 9000 residents live on the campus of HIT (second campus, approximately 0.35 km^2^).

Twenty-four-hour PM_1.0_ and PM_2.5_ sampling was conducted every 7 days during the normal periods and every day during the polluted days from Nov. 2014 to Feb. 2015, using a fine particulate dust sampler (TH-150CIII and TH-150A, Mingxuan Company, Chengdu, China). The fine particulate dust sampler ran at a constant flow rate of 100 L/min. The 47-mm Teflon filters, with low inherent contaminants, were purchased from Pall Company (USA) and were dried/balanced to a constant weight (accuracy of 0.00001 g) at 298 K for 24 h before sampling. Filters were handled with tweezers coated with Teflon tape to reduce the possibility of contamination. In total, more than 150 samples were collected during the whole observation period. Number concentrations of PM_1.0_ and PM_2.5_ were collected by hand-held number concentration meters (TSI Company, USA), accompanied by the sampling of fine particles. Samples were taken three times a day (8:00 am., 12:00 am and 4:00 pm), each time for 5 min.

Chemical analysis. After weighing, the filters were sectioned, and one-fourth of the filter was cut into small pieces and soaked in a 60 mL polytetrafluoroethylene (PTFE) vial. The WSIs of the PM were obtained by ultrasonicating the filters for 150 min in 50 mL deionized water and then filtering the water extracts through a 0.45-μm cellulose acetate filter before chemical ion analysis. Concentrations of the cations (K^+^, Na^+^, Mg^2+^, Ca^2+^) were analyzed by inductively coupled plasma-atomic emission spectrometry (ICP-AES); the anions (SO_4_^2−^, NO_3_^−^, Cl^−^, F^−^) were determined using an ion chromatograph (Dionex 4500i, USA); and NH_4_^+^ was determined by Nessler’s reagent spectrophotometry.

To detect the species concentration distribution of the heavy metals within the atmospheric PM, one-quarter of the sampling filters was cut into small pieces, and the metals within the PM were extracted and analyzed according to Hu *et al*.^16^. Briefly, one-quarter of the sampling filters was cut into small pieces and immersed in a 15 mL digestion solution of concentrated HNO_3_ and concentrated HClO_4_ (3:1) for 10 h in a fume hood. Then, the beaker was baked with the temperature maintained at 100 °C until white smoke appeared, and the temperature was increased to 150 °C to volatilize the acid. When the sample solution had evaporated to near dryness, 2 mL of 2% nitric acid was added three times with continued heating. After being digested and cooled, the remaining solution was transferred to a 50 mL volumetric flask. In the next step, the flask was brought to the final volume by employing 2% HNO_3_. After filtration, the filtrate was stored in a 15-mL centrifugal tube and subjected to ICP-OES (VISTAMPX, US) for Al, Ca, Fe, K, Mg, Na, S and Si detection and ICP-mass spectrometry (MS) (VG PQ ExCell, Thermo Fisher Scientific Inc., USA) for Ti, Cr, Mn, Ni, Cu, Zn, As, Sr, Ba and Pb analysis.

Chemical–mineralogical speciation of heavy metals in the collected PM_1.0_ and PM_2.5_ samples was operatively measured by a five-step sequential chemical extraction procedure, which followed the extraction methods proposed by Tessier *et al*.^20^ Specifically, sequential chemical extraction was carried out with 50 mL polypropylene centrifuge bottles. The water-exchangeable metal species were extracted with 20 mL MgCl_2_ (1 M, pH 7.0) and with oscillation at 25 ± 5 °C for 16 h, after which the extracted solutions were separated from the filters by centrifugation at 4000 rpm at 25 °C for 10 min. Similarly, the acid-exchangeable, reducible, oxidizable and residual heavy metal fractions were extracted using NaOAC (1 M, pH 5.0), NH_2_OH·HCl (0.04 M), H_2_O_2_ (8.8 M) and HF-HClO_4_ mixtures, respectively. The combined supernatants were heated until 1–2 mL of solution remained, and then they were diluted to a volume of 10 mL with 2% HNO_3_ to be stored in a polyethylene bottle at 4 °C before analysis.

### Source apportionment analysis

To understand the probable contributions from local point sources, PCA-MLR and the chemical mass balance (CMB) model were applied for air pollution source apportionment, according to the previous studies of Shi *et al*.^35^ and Bhuyan *et al*.^34^. The CMB model was freely available for use on the US EPA official website. Seasonal pollution sources of PM_2.5_ and PM_1.0_ were analyzed and measured (Mar., Apr. and May were regarded as spring; Jun., Jul. and Aug. as summer; Sep., Oct. and Nov. as autumn; and Dec., Jan. and Feb. as winter).

### Risk assessment

The risk assessment model developed by the US EPA was applied to evaluate the health risks posed by heavy metals in PM_1.0_ and PM_2.5_. Considering the variety of physiological characteristics and lifestyles of Harbin City residents, we divided them into three groups: male (>16 years), female (>16 years) and children (<16 years). Since metal exposure can occur through direct inhalation, ingestion, and dermal contact, the average daily dose (in mg·kg^−1^·d^−1^) through ingestion (*ADD*_ing_), inhalation (*ADD*_inh_), and dermal contact (*ADD*_derm_) can be calculated as follows^27,44^:

3 $${{\rm{ADD}}}_{{\rm{Ing}}}=\frac{{\rm{C}}\times {\rm{IngR}}\times {\rm{CF}}\times {\rm{EF}}\times {\rm{ED}}}{{\rm{BW}}\times {\rm{AT}}}$$

4 $${{\rm{ADD}}}_{{\rm{Inh}}}=\frac{C\times {\rm{Inh}}R\times {\rm{EF}}\times {\rm{ED}}}{{\rm{PEF}}\times {\rm{BW}}\times {\rm{AT}}}$$

5 $${{\rm{ADD}}}_{{\rm{derm}}}=\frac{{\rm{C}}\times {\rm{SA}}\times {\rm{CF}}\times {\rm{SL}}\times {\rm{ABS}}\times {\rm{EF}}\times {\rm{ED}}}{{\rm{BW}}\times {\rm{AT}}}$$

where *C* stands for the concentration of the contaminant in PM_2.5_ (mg/kg for *ADD*_ing_ and *ADD*_derm_, μg/m^3^ for *ADD*_inh_). For ingestion, the intake rate (*IngR*) was 100, 100 and 200 mg/day for males, females and children, respectively. For inhalation, Inh*R* was 15.2 m^3^/day for males. *BW*, the average body weight, was 62.7, 54.4 and 15 kg for males, females and children, respectively. *SA*, the surface area of the skin that contacts the airborne particles (cm^2^), was 4220, 3820 and 2160 for males, females and children, respectively. *AF* is the skin adherence factor for airborne particulates (mg/cm^2^), which was 0.07, 0.07 and 0.2 for males, females and children, respectively. *PEF* refers to the particle emission factor and was 1.36 × 10^9^ m^3^/kg. *SL* is the skin adherence factor, equal to 0.2 mg/cm^2^d. *EF* is the exposure frequency, in days/year, and was 180 days. *ED* is the exposure duration in years, equal to 24, 24 and 6 years for males, females and children, respectively. *ET* stands for exposure time, 24 h/day. *AT* refers to the averaging time in days, equal to *ED* × 365. *ABS* refers to the dermal absorption factor, which was 0.001 for Cd, and 0.01 for the other metals. *CF*, the conversion factor (kg/mg), is 10^–6^.

## Supplementary information

Supplementary information.

## Data Availability

The datasets generated and/or analyzed during the current study are available from the corresponding author upon reasonable request.

## References

[1] Lelieveld J, Evans JS, Fnais M, Giannadaki D, Pozzer A (2015). The contribution of outdoor air pollution sources to premature mortality on a global scale. Nature..

[2] Liu MM (2017). Spatial and temporal trends in the mortality burden of air pollution in China: 2004-2012. Environ Int..

[3] Hu W (2014). Personal and indoor PM2.5 exposure from burning solid fuels in vented and unvented stoves in a rural region of china with a high incidence of lung cancer. Environ Sci Technol..

[4] Loomis D, Huang W, Chen GS (2014). The International Agency for Research on Cancer (IARC) evaluation of the carcinogenicity of outdoor air pollution: focus on China. Chin J Cancer..

[5] Xing YF, Xu YH, Shi MH, Lian YX (2016). The impact of PM2.5 on the human respiratory system. J Thorac Dis..

[6] Kreyling WG, Semmler-Behnke M, Moller W (2006). Health implications of nanoparticles. J Nanopart Res..

[7] Yang, J., Xia, L. I., Qin, L. I. & Wang, Z. W. Variation characteristics of atmospheric stability and mixed layer thickness and their relation to air pollution in recent 30 years in Urumqi. Arid Land Geography. (2011).

[8] Zhang RY (2015). Formation of urban fine particulate matter. Chem Rev..

[9] Hao Y (2020). Quantification of primary and secondary sources to PM2.5 using an improved source regional apportionment method in an industrial city. China. Sci Total Environ..

[10] Zhang Q, He KB, Huo H (2012). Cleaning China’s air. Nature..

[11] Zheng SM, Yi HT, Li H (2015). The impacts of provincial energy and environmental policies on air pollution control in China. Renew Sust Energ Rev..

[12] Liu T (2016). Formation of secondary aerosols from gasoline vehicle exhaust when mixing with SO2. Atmos Chem Phys..

[13] Fu HB, Chen JM (2017). Formation, features and controlling strategies of severe haze-fog pollutions in China. Sci Total Environ..

[14] Tao MH (2014). Formation process of the widespread extreme haze pollution over northern China in January 2013: Implications for regional air quality and climate. Atmos Environ..

[15] Du H (2011). Insights into summertime haze pollution events over Shanghai based on online water-soluble ionic composition of aerosols. Atmos. Environ..

[16] Sun YL (2014). Investigation of the sources and evolution processes of severe haze pollution in Beijing in January 2013. J. Geophys. Res. Atmos..

[17] Cheng HR (2014). Ionic composition of submicron particles (PM1.0) during the long lasting haze period in January 2013 in Wuhan, Central China. J. Environ. Sci. (China).

[18] Gao JJ (2015). The variation of chemical characteristics of PM2.5 and PM10 and formation causes during two haze pollution events in urban Beijing, China. Atmos Environ..

[19] Wei LL (2017). Adsorption of Cu2+ and Zn2+ by extracellular polymeric substances (EPS) in different sludges: Effect of EPS fractional polarity on binding mechanism. J Hazard Mater..

[20] Hu X (2012). Bioaccessibility and health risk of arsenic and heavy metals (Cd, Co, Cr, Cu, Ni, Pb, Zn and Mn) in TSP and PM2.5 in Nanjing, China. Atmos Environ..

[21] Wu Y (2019). Seasonal variations, source apportionment, and health risk assessment of heavy metals in PM2.5 in Ningbo, China. Aerosol Air Qual Res..

[22] Cui, L. M. *et al*. Chemical content and source apportionment of 36 heavy metal analysis and health risk assessment in aerosol of Beijing. Environ Sci Pollut R. 10.1007/s11356-019-06427-w (2019). 10.1007/s11356-019-06427-w 31879890

[23] Zhai YB (2014). Source identification and potential ecological risk assessment of heavy metals in PM2.5 from Changsha. Sci Total Environ..

[24] Tessier A (1979). Sequential extraction procedure for the speciation of particulate trace metals. Analytical chemistry..

[25] Xue S (2008). Trihalomethane formation potential of organic fractions in secondary effluent, J. Environ. Sci. -Chin..

[26] Jones AM, Harrison RM (2004). The effects of meteorological factors on atmospheric bioaerosol concentrations - a review. Sci Total Environ..

[27] Fang WX (2013). PM10 and PM2.5 and health risk assessment for heavy metals in a typical factory for cathode ray tube television recycling. Environ Sci Technol..

[28] Lee BK, Hieu NT (2011). Seasonal variation and sources of heavy metals in atmospheric aerosols in a residential area of Ulsan, Korea. Aerosol Air Qual Res..

[29] Han YJ (2008). Ionic constituents and source analysis of PM2.5 in three Korean cities. Atmos Environ..

[30] Deshmukh DK (2011). Water soluble ions in PM2.5 and PM1.0 aerosols in Durg city, Chhattisgarh, India. Aerosol Air Qual Res..

[31] Hassanvand MS (2014). Indoor/outdoor relationships of PM10, PM2.5, and PM1.0 mass concentrations and their water-soluble ions in a retirement home and a school dormitory. Atmos Environ..

[32] Prakash J (2018). Chemical characterization and quantitativ e assessment of source-specific health risk of trace metals in PM1.0 at a road site of Delhi, India. Environ Sci Pollut R..

[33] Sun YL (2006). Chemical characteristics of PM2.5 and PM10 in haze-fog episodes in Beijing. Environ Sci Technol..

[34] Bhuyan P (2018). Chemical characterization and source apportionment of aerosol over mid Brahmaputra Valley, India. Environ Pollut..

[35] Cusack M (2013). Variability of sub-micrometer particle number size distributions and concentrations in the Western Mediterranean regional background. Tellus B..

[36] Shi GL (2011). Estimated contributions and uncertainties of PCA/MLR-CMB results: source apportionment for synthetic and ambient datasets. Atmos Environ..

[37] Shang Y (2019). Cytotoxicity comparison between fine particles emitted from the combustion of municipal solid waste and biomass. J Hazard Mater..

[38] Sabiene N (2008). Variations of metal distribution in sewage sludge composting. Waste Manage..

[39] Zhang Y (2014). Solid Phase Extraction of Mn, Co, Cr, Zn, and Pb on titanium dioxide nanotubes for their determination by inductively coupled plasma mass spectrometry. Atom Spectrosc..

[40] Ma LQ (1997). Chemical fractionation of cadmium, copper, nickel, and zinc in contaminated soils. J Environ Qual..

[41] Yap CK (2002). Concentrations of Cu and Pb in the offshore and intertidal sediments of the west coast of Peninsular Malaysia. Environ Int..

[42] Zheng W (2010). Study on mechanisms and effect of surfactant-enhanced air sparging. Water Environ Res..

[43] Yan HY (2016). Influence of outdoor temperature on the indoor environment and thermal adaptation in Chinese residential buildings during the heating season. Energ. Buildings..

[44] Office of Research and Development, United States Environmental Protection Agency (U.S. EPA). Risk Characterization Equations; U.S. EPA: Washington, D.C. (1997).

