# Supplementary material for: Seasonal concentration distribution of PM1.0 and PM2.5 and a risk assessment of bound trace metals in Harbin, China: Effect of the species distribution of heavy metals and heat supply
Source: Sci Rep. 2020 May 18;10:8160. doi: 10.1038/s41598-020-65187-7 (PMC7235082; doi:10.1038/s41598-020-65187-7)
Supplement: Supplementary file 1 — Supplementary information. [file 41598_2020_65187_MOESM1_ESM.docx]

**Supplementary Materials**

**Seasonal concentration distribution of PM_1.0_ and PM_2.5_ and a risk assessment of bound trace metals in Harbin, China: Effect of the species distribution of heavy metals and heat supply**

**Kun Wang^1^, Weiye Wang^1^, Lili Li^1^, Jianju Li^1^, Liangliang Wei^1,^**^∗^**, Wanqiu Chi^1^, Lijing Hong^1,2^, Qingliang Zhao^1^, Junqiu Jiang^1^**

1. State Key Laboratory of Urban Water Resources and Environment; School of Environment, Harbin Institute of Technology, Harbin 150090, China.

2. Suzhou Industrial Park Design & Research Co., Ltd, Suzhou, 215000

∗weill333@163.com

Table S1 Ratio of the mass concentration and particle number of PM_1.0_ in each month

| Month | Nov. | Dec. | Jan. | Feb. | Mar. | Apr. | May | Jun. | Jul. | Aug. | Sep. | Oct. |
| --- | --- | --- | --- | --- | --- | --- | --- | --- | --- | --- | --- | --- |
| Ratio | 433 | 618 | 366 | 423 | 371 | 206 | 507 | 483 | 951 | 866 | 1002 | 269 |

Table S2 Concentrations of inorganic/metal elements and WSIs in PM_2.5_ in different months from Nov. 2014 to Oct. 2015 (unit: μg/m^3^)

|  |  | Dec. | Jan. | Feb. | Mar. | Apr. | May | Jun. | Jul. | Aug. | Sep. | Oct. | Nov. |
| --- | --- | --- | --- | --- | --- | --- | --- | --- | --- | --- | --- | --- | --- |
| Al | Inorganic  /metal | 0.67 | 0.49 | 2.00 | 0.82 | 1.73 | 1.25 | 0.40 | 0.18 | 0.15 | 0.34 | 0.41 | 0.38 |
| Fe |  | 2.29 | 1.75 | 2.95 | 1.52 | 3.43 | 2.35 | 4.02 | 4.08 | 1.35 | 2.31 | 1.75 | 0.79 |
| S |  | 7.57 | 17.32 | 11.91 | 5.24 | 3.00 | 14.62 | 2.82 | 2.17 | 1.40 | 3.18 | 4.15 | 4.88 |
| Si |  | 0.66 | 6.54 | 5.14 | 1.07 | 0.97 | 5.84 | 0.79 | 0.94 | 0.77 | 1.12 | 0.84 | 0.86 |
| Zn |  | 0.53 | 0.56 | 0.48 | 0.80 | 0.59 | 0.52 | 0.33 | 0.45 | 0.26 | 0.47 | 0.59 | 0.80 |
| Ti |  | 0.31 | 0.40 | 0.40 | 0.42 | 0.43 | 0.40 | 0.34 | 0.48 | 0.34 | 0.47 | 0.57 | 0.41 |
| Pb |  | 0.21 | 0.18 | 0.25 | 0.29 | 0.15 | 0.22 | 0.19 | 0.11 | 0.09 | 0.13 | 0.15 | 0.09 |
| As |  | 0.08 | 0.04 | 0.03 | 0.03 | 0.04 | 0.03 | 0.01 | 0.01 | 0.00 | 0.01 | 0.02 | 0.02 |
| Ba |  | 0.10 | 0.11 | 0.30 | 0.18 | 0.11 | 0.20 | 0.03 | 0.06 | 0.02 | 0.03 | 0.05 | 0.03 |
| Mn |  | 0.06 | 0.04 | 0.06 | 0.04 | 0.15 | 0.05 | 0.13 | 0.08 | 0.05 | 0.05 | 0.05 | 0.05 |
| Cu |  | 0.07 | 0.10 | 0.13 | 0.11 | 0.14 | 0.11 | 0.04 | 0.04 | 0.04 | 0.11 | 0.18 | 0.05 |
| Cr |  | 0.08 | 0.12 | 0.20 | 0.12 | 0.08 | 0.16 | 0.41 | 0.54 | 0.14 | 0.25 | 0.18 | 0.12 |
| Sr |  | 0.03 | 0.02 | 0.08 | 0.05 | 0.04 | 0.05 | 0.01 | 0.01 | 0.01 | 0.01 | 0.01 | 0.02 |
| Ni |  | 0.01 | 0.08 | 0.03 | 0.03 | 0.04 | 0.05 | 0.03 | 0.08 | 0.05 | 0.04 | 0.05 | 0.06 |
| Ca |  | 10.59 | 6.74 | 8.44 | 9.26 | 15.59 | 7.59 | 5.72 | 6.72 | 3.68 | 4.72 | 6.57 | 6.90 |
| K |  | 2.6385 | 1.54707 | 4.3565 | 3.2152 | 1.8281 | 2.9518 | 1.3632 | 1.2729 | 0.8973 | 2.3794 | 5.2281 | 5.0503 |
| Mg |  | 0.9549 | 0.56078 | 1.3488 | 1.0082 | 1.6493 | 0.9548 | 0.5116 | 1.6777 | 0.3612 | 0.6535 | 0.9708 | 0.9137 |
| Na |  | 14.018 | 14.6779 | 8.3928 | 7.6559 | 12.057 | 11.535 | 6.4926 | 7.6433 | 5.0361 | 10.774 | 12.903 | 8.4289 |
| Ca^2+^ | WSIs | 2.9545 | 10.5465 | 3.0075 | 2.8495 | 3.986 | 1.5409 | 1.1795 | 1.111 | 1.0113 | 1.2494 | 1.7976 | 1.8735 |
| K^+^ |  | 1.463 | 1.0995 | 1.697 | 1.8175 | 1.198 | 0.9801 | 0.7234 | 0.592 | 0.5111 | 0.8526 | 2.9511 | 2.3195 |
| Mg^2+^ |  | 0.1165 | 0.476 | 0.909 | 0.446 | 0.377 | 0.1807 | 0.1417 | 0.236 | 0.1103 | 0.254 | 0.4383 | 0.1795 |
| Na^+^ |  | 1.5935 | 0.392 | 1.664 | 0.69 | 5.736 | 2.5289 | 1.3992 | 2.21 | 1.2041 | 2.7679 | 5.2312 | 1.3705 |
| F^-^ |  | 0.1935 | 0.24 | 0.4915 | 0.354 | 0.3025 | 0.0648 | 0.0273 | 0.1389 | 0.0377 | 0.057 | 0.2207 | 0.249 |
| NH_4_^+^ |  | 0.2605 | 0.346 | 0.5855 | 0.5105 | 0.081 | 0.0814 | 0.0431 | 0.0945 | 0.0672 | 0.097 | 0.2477 | 0.287 |
| Cl^-^ |  | 3.5145 | 4.3535 | 13.1 | 7.285 | 0.396 | 1.8443 | 0.8437 | 0.4565 | 0.3348 | 0.24 | 2.696 | 5.462 |
| SO_4_^2-^ |  | 12.954 | 33.882 | 17.133 | 27.08 | 2.169 | 5.298 | 5.3682 | 2.1972 | 2.1399 | 4.575 | 4.5904 | 11.148 |
| NO_3_^-^ |  | 8.4605 | 18.5025 | 22.355 | 7.754 | 1.630 | 3.022 | 5.0166 | 1.9287 | 1.332 | 1.184 | 7.8234 | 12.839 |
| Σ |  | 72.39 | 121.12 | 107.44 | 80.64 | 57.90 | 64.43 | 38.37 | 35.49 | 21.41 | 38.31 | 60.66 | 65.58 |

Table S3 Concentration ratios of the inorganic/metal elements in PM_1.0_ and PM_2.5_ in four seasons

|  | Spring | Summer | Autumn | Winter |
| --- | --- | --- | --- | --- |
|  | PM_1.0/_PM_2.5_ | PM_1.0/_PM_2.5_ | PM_1.0/_PM_2.5_ | PM_1.0/_PM_2.5_ |
| Al | 0.70 | 0.80 | 0.72 | 0.92 |
| Ca | 0.91 | 0.84 | 0.88 | 0.90 |
| Fe | 0.85 | 0.76 | 0.83 | 0.80 |
| K | 0.81 | 0.85 | 0.88 | 0.77 |
| Mg | 0.82 | 0.63 | 0.80 | 0.87 |
| Na | 0.90 | 0.93 | 0.87 | 0.81 |
| S | 0.48 | 0.88 | 0.90 | 0.70 |
| Si | 0.31 | 0.80 | 0.77 | 0.72 |
| Zn | 0.84 | 0.84 | 0.88 | 0.90 |
| Ti | 0.93 | 0.95 | 0.90 | 0.98 |
| Pb | 0.63 | 0.71 | 0.85 | 0.81 |
| As | 0.99 | 0.67 | 0.90 | 0.85 |
| Ba | 0.68 | 0.73 | 0.83 | 0.65 |
| Mn | 0.88 | 0.67 | 0.86 | 0.88 |
| Cu | 0.59 | 0.63 | 0.58 | 0.58 |
| Cr | 0.85 | 0.79 | 0.88 | 0.87 |
| Sr | 0.76 | 0.93 | 0.87 | 0.79 |
| Ni | 0.67 | 0.58 | 0.58 |  |

Fig. S1 Number of PM_1.0_ particles and the average humidity and temperature in different months from Nov. 2014 to Oct. 2015


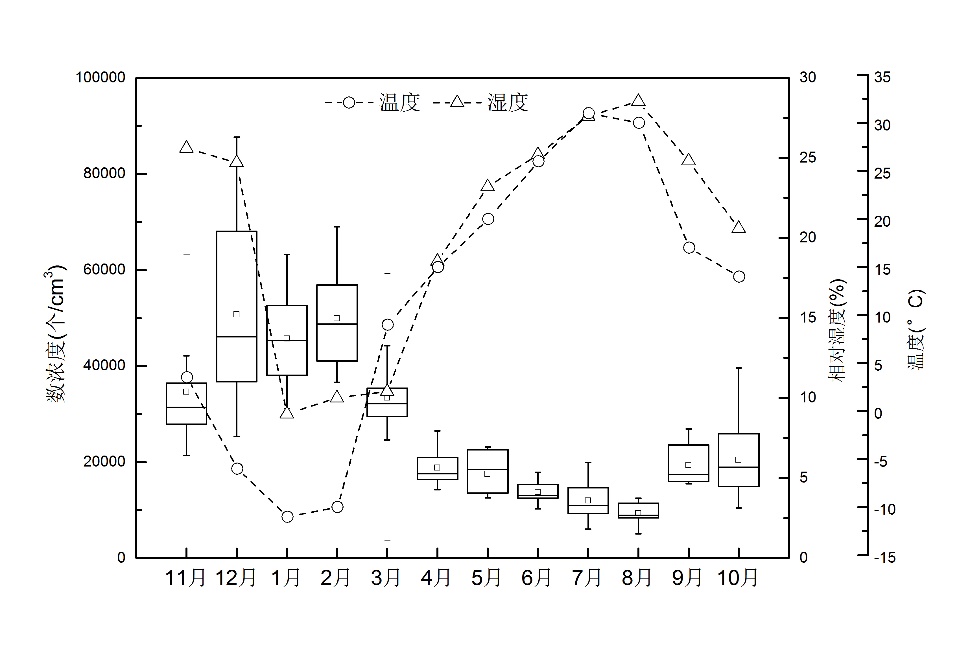


Humid.

Temp.

Nov. Dec. Jan. Feb. Mar. Apr. May Jun. Jul. Aug. Sep. Oct.

Particles number (cm^-3^)

Humidity (%)

Temperature (^o^C)

Temperature (^o^C)

Fig. S2 Characteristics variation in the inorganic/metal elements in PM_2.5_ (a) and PM_1.0_ (b) among different seasons

|  |  |  |  |
| --- | --- | --- | --- |
| **a**  Spring | Summer | Autumn | Winter |

|  |  |  |  |
| --- | --- | --- | --- |
| **b**  Spring | Summer | Autumn | Winter |
|  | | | |
